# Integrative Analysis of Metabolomic and Transcriptomic Profiles Uncovers Biological Pathways of Feed Efficiency in Pigs

**DOI:** 10.3390/metabo10070275

**Published:** 2020-07-06

**Authors:** Priyanka Banerjee, Victor Adriano Okstoft Carmelo, Haja N. Kadarmideen

**Affiliations:** Quantitative Genomics, Bioinformatics and Computational Biology Group, Department of Applied Mathematics and Computer Science, Technical University of Denmark, 2800 Kongens Lyngby, Denmark; priyankabnrj@gmail.com (P.B.); vaocar@dtu.dk (V.A.O.C.)

**Keywords:** feed efficiency, linear model, metabolomics, pigs, transcriptomics

## Abstract

Feed efficiency (FE) is an economically important trait. Thus, reliable predictors would help to reduce the production cost and provide sustainability to the pig industry. We carried out metabolome-transcriptome integration analysis on 40 purebred Duroc and Landrace uncastrated male pigs to identify potential gene-metabolite interactions and explore the molecular mechanisms underlying FE. To this end, we applied untargeted metabolomics and RNA-seq approaches to the same animals. After data quality control, we used a linear model approach to integrate the data and find significant differently correlated gene-metabolite pairs separately for the breeds (Duroc and Landrace) and FE groups (low and high FE) followed by a pathway over-representation analysis. We identified 21 and 12 significant gene-metabolite pairs for each group. The valine-leucine-isoleucine biosynthesis/degradation and arginine-proline metabolism pathways were associated with unique metabolites. The unique genes obtained from significant metabolite-gene pairs were associated with sphingolipid catabolism, multicellular organismal process, cGMP, and purine metabolic processes. While some of the genes and metabolites identified were known for their association with FE, others are novel and provide new avenues for further research. Further validation of genes, metabolites, and gene-metabolite interactions in larger cohorts will elucidate the regulatory mechanisms and pathways underlying FE.

## 1. Introduction

Feed represents about 60–70% of total pork production costs in modern pig production. Thus, to decrease costs and increase profitability, it is pivotal to identify feed efficient (FE) animals [1]. However, due to the polygenic architecture of FE, individual pigs in a herd exhibit considerable variation in FE despite belonging to similar genetic background and environment [2]. Considering this variation, different methods have been proposed and widely used to measure the FE, including feed conversion ratio (FCR) and residual feed intake (RFI) [1,3]. FCR is the ratio of feed intake (FI) per unit body weight gain and is affected by many factors such as breed, sex, diet, and environmental conditions [4,5]. Pigs with low FCR are considered high FE and vice-versa. RFI estimates the difference between actual and expected FI predicted on production traits as average daily gain (ADG) [6]. Since FCR considers both FI and weight gain, and FCR is also one of the critical predictors of FE, it suggests that feed efficient pigs may possess different physiological-biochemical profiles compared to the inefficient ones [2].

Based on the advances in omics technologies, several approaches have been adopted to shed light on the genetic mechanisms underlying FE in pigs. Among these omics technologies, transcriptomics and metabolomics have provided tools to elucidate the molecular basis of FE. While transcriptomics allows us to have a transcriptional snapshot of genes underpinning the phenotype under investigation, metabolomics bridges the gap between genotype and phenotype. Recently, an increasing number of metabolomic studies have reported the role of metabolites in economically important traits [7], such as meat quality [8,9], pre-slaughter stress [10], and FE [3]. Likewise, several transcriptomic studies have pointed out candidate genes underpinning FE and other related-traits such as immune response, growth, and metabolism in pigs [11,12,13].

Recently, we have investigated RNA-seq data on the 41 Danish production pigs that underwent feed efficiency and performance testing trials to identify differentially expressed genes and gene networks and reported 13 genes as potential biomarkers for feed efficiency [14]. Despite the new insights into key genes and molecular mechanisms reported in these studies, these approaches rely solely on data from a single biological layer. It has been shown that the integration of transcriptomics datasets with genomic and other omics datasets (systems genomics) increases the power to detect causal and regulatory factors and molecular pathways underlying complex phenotypes or diseases in animals [15,16]. 

To gain further insights into biochemical aspects of complex traits, data integration analysis has emerged as a fruitful tool, unveiling potential biomarkers via integration of metabolomics and transcriptomics [17]. By the development of analytical technologies for data integration, the assessment of system-wide changes of transcript levels as surrogate measurements of metabolic rearrangements can be widely assessed. Metabolite-transcript co-responses using combined profiling can yield vital information on the complex biological regulation of the trait. Transcriptome-metabolome integration is a powerful combination as the metabolome is affected by the phenotypic measurements to which the global measures of transcriptome can be anchored [18]. Therefore, herein, we integrated data from metabolome-transcriptome approaches to unveil the unique gene-metabolite pairs. To this end, we adopted a two-step framework, as follows: (1) we first employed the numerical integration of gene-metabolite levels to identify gene-metabolite interaction pairs separately for the breeds (Duroc and Landrace) and FE groups (low and high FE) using IntLIM R-package; (2) next, a knowledge-based integration approach based on pathway over-representation analysis was used to reveal the underlying pathways in each group (breed-specific and FE-specific). To the best of our knowledge, this is the first study of its kind to ever combine high throughput metabolomics data with RNA sequence based gene expression data in pigs to unravel the missing links between genes and metabolites and to shed light on the molecular basis that characterizes the specific differences based on breed and feed efficiency.

## 2. Results

### 2.1. Descriptive Statistics and Linear Model Analysis for Genes and Metabolites

**Figure 1:**
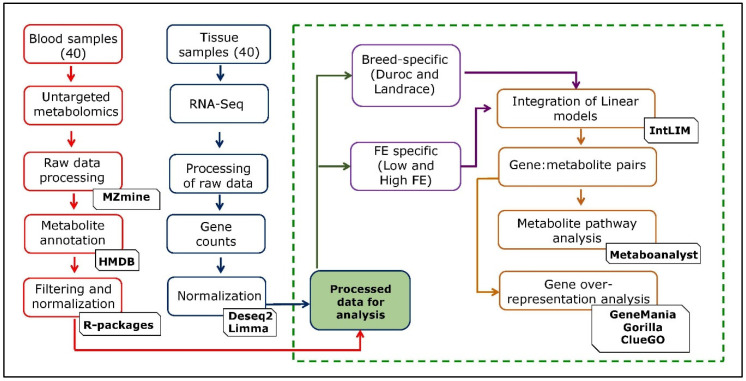
Schematic representation of the study design and analysis steps.

The data on 749 metabolites and 25,880 genes from 40 samples were generated using untargeted metabolomics and transcriptomic approaches, respectively. We utilized data of 405 annotated metabolites (see methodology) for further analysis. For the transcriptomic data generated on 25,880 genes, we analyzed the data for each of the two groups (breed-specific and FE-specific), as described in the methodology. The genes with a gene count <1 were removed, resulting in 20,233 genes for both the groups. The gene count data for each group (breed-specific and FE-specific) was normalized, and the linear model was fitted into the data as given in the methodology. The genes were also screened for their chromosomal information from the Ensembl *Sus scrofa* database. After normalization, removal of values < 0 and obtaining the gene chromosomal location information, 17,726 (breed-specific), and 17,697 (FE-specific) genes were retained in each group. As a quality control for IntLIM, we filtered out genes with the lowest 5% of the variation, which gave 16,839 genes (breed-specific) and 16,812 genes (FE-specific) that were subjected to IntLIM analysis. A schematic representation of the study design and analysis steps are given in Figure 1. We performed the PCA analysis (Figure S1) on the filtered metabolome-transcriptome data, which included 405 metabolites, and 16,839 genes (breed-specific), and 16,812 genes (FE-specific). The results of PCA analysis for the metabolites-genes based on breed and FE groups are shown in Figure S1.

### 2.2. Gene Metabolite Interaction of Breed-Specific and FE-Specific Groups

**Figure 2:**
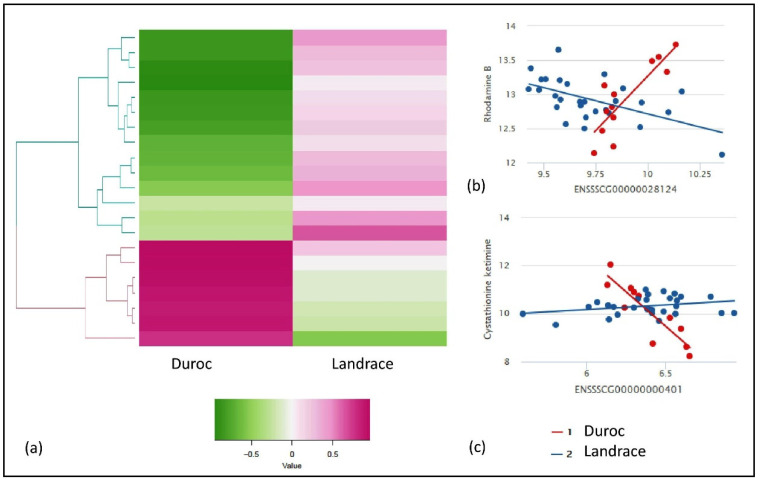
Results of IntLIM applied to breed-specific groups. (**a**) Clustering of 21 identified gene-metabolite pairs (FDR adjusted *p*-value of interaction coefficient < 0.1, Spearman correlation difference > 0.1 in Duroc and Landrace breeds, (**b**) Gene-metabolite difference in ENSSSCG00000028124 (*SNRPN*)—rhodamine B (FDR adjusted *p*-value = 0.1, Duroc Spearman correlation = 0.7, Landrace Spearman correlation = −0.5), (**c**) Gene-metabolite difference in ENSSSCG00000000401 (*GLS2*)—cystathionine ketimine (FDR adjusted *p*-value = 0.01, Duroc Spearman correlation = −0.9, Landrace Spearman correlation = 0.2).

From the IntLIM analysis, we identified gene-metabolite pairs that have a strong association with respect to the breed (Duroc and Landrace) and FE (low and high FE) groups. For the breed-specific groups, all possible combinations of gene-metabolite pairs (6,819,795 model runs) were evaluated, using Duroc and Landrace as the breed-group. Based on this approach, we identified 21 gene-metabolite associations (false discovery rate—FDR adjusted *p*-value ≤ 0.1 and correlation difference effect size > 0.1) (Table 1). Clustering these pairs by the direction of association (positive and negative correlations) within each breed group revealed two major clusters (Figure 2a) in each breed. First, the Duroc correlated/Landrace anti-correlated cluster consists of seven gene-metabolite pairs (three unique metabolites and five unique genes) with a high positive correlation in Duroc and low or negative correlation in Landrace (Figure 2a). Second, the Duroc anti-correlated/Landrace correlated cluster consists of 14 gene-metabolite pairs (10 unique metabolites and nine unique genes) with relatively high negative correlations in Duroc and positive correlations in Landrace. The two top-ranked gene-metabolite pairs (ranked in descending order of absolute value of Spearman correlation difference between Duroc and Landrace) in the Duroc correlated and anti-correlated clusters were ENSSSCG00000028124 (*SNRPN*)—Rhodamine B (Figure 2b) and ENSSSCG00000000401 (*GLS2*)—cystathionine ketimine (Figure 2c) respectively. *SNRPN* and Rhodamine B are positively correlated in Duroc (r = 0.7) but negatively correlated in Landrace (r = −0.5) (Figure 2b). *GLS2* and cystathionine ketimine are negatively correlated in Duroc (r = −0.9), but positively correlated in Landrace (r = 0.2) (Figure 2c).

**Figure 3:**
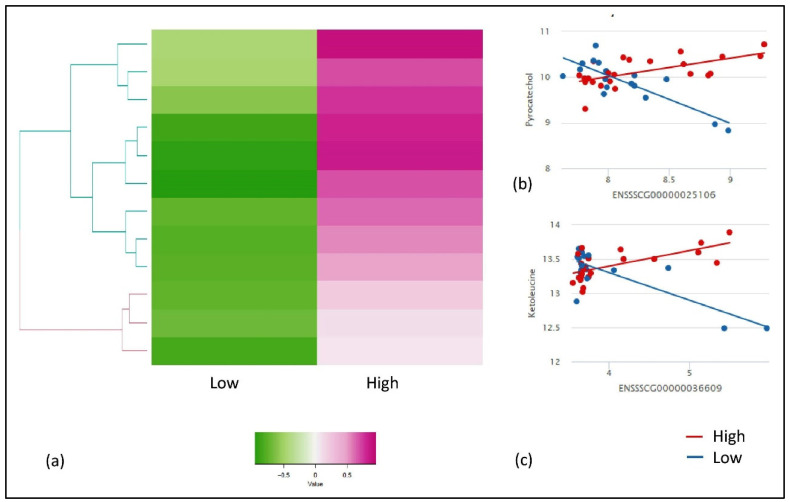
Results of IntLIM applied to FE-specific groups. (**a**) Clustering of 12 identified gene-metabolite pairs (FDR adjusted *p*-value of interaction coefficient < 0.1, Spearman correlation difference > 0.1 in high and low FE groups, (**b**) Gene-metabolite difference in ENSSSCG00000025106 (*THNSL2*)—Pyrocatechol (FDR adjusted *p*-value = 0.06, High-FE Spearman correlation = 0.6, Low-FE Spearman correlation = −0.7), (**c**) Gene-metabolite difference in ENSSSCG00000036609 (*TBXT*)—ketoleucine (FDR adjusted *p*-value = 0.08, High-FE Spearman correlation = 0.5, Low-FE Spearman correlation = −0.3).

Similarly, we used IntLIM for the FE-specific group and evaluated all possible combinations of gene-metabolite pairs (6,808,860 models), with low and high FE as a binary phenotype. With this approach, we identified 12 FE-specific gene-metabolite correlations (FDR adjusted interaction *p*-value ≤ 0.1, and a Spearman correlation difference > 0.1) involving eight unique gene and metabolites each (Table 2). The heat map with gene-metabolite Spearman correlation for low and high FE group showed a clear separation between the two groups (Figure 3a). The high FE-correlated cluster of 12 gene-metabolite pairs (eight unique genes and metabolites with high correlations in high-FE groups) were negatively correlated with the low-FE group. The two gene-metabolite pairs (ranked in descending order of absolute value Spearman correlation difference between high and low FE group) in high-FE correlated clusters were ENSSSCG00000025106 (*THNSL2*)—pyrocatechol (Figure 3b) and ENSSSCG00000036609 (*TBXT*)—ketoleucine (Figure 3c), respectively. Both pairs showed a positive correlation in high-FE group (r = 0.6, r = 0.5) while showed a negative correlation in the low-FE group (r = −0.7, r = −0.3) (Figure 3b,c).

### 2.3. Pathway and Gene Ontology Over-Representation Analysis

We identified the pathways associated with the unique metabolites in each cluster identified from breed-specific (21) and FE-specific (12) interactions. The three unique metabolites from Duroc correlated/Landrace anti-correlated clusters were associated with arginine and proline metabolism (*p*-value = 0.02). Furthermore, the ten unique metabolites from Duroc anti-correlated/Landrace correlated cluster were associated with valine-leucine-isoleucine biosynthesis (*p*-value = 0.01) and valine-leucine-isoleucine degradation (*p*-value = 0.07) along with arginine and proline metabolism (*p*-value = 0.07). The eight unique metabolites from high-FE correlated/low-FE anti-correlated cluster were associated with valine-leucine-isoleucine biosynthesis (*p*-value = 0.01) and degradation (*p*-value = 0.07). The pathways associated with the metabolites in breed-specific and FE-specific clusters for unique metabolites are given in Supplementary Table S1a. 

Unique and mappable genes from each group (breed-specific—each cluster, and FE-specific) were screened by using GeneMania to generate a composite functional association network that includes all the evidence of co-functionality. From the breed-specific group, unique genes (4 genes) from Duroc correlated/Landrace anti-correlated cluster (Table 1) mapped to 20 genes based on co-functionality from GeneMania (Table S1b). The gene-ontology enrichment analysis of the identified 24 genes (unique genes from Table 1 and co-functional genes from GeneMania) revealed enrichment of the regulation of hemopoiesis, response to thyroid hormone, and the sphingolipid catabolic process (Table S1c). These genes were enriched for the sphingolipid metabolism KEGG pathway (adjusted *p*-value corrected with Bonferroni step down ≤ 0.05) (Supplementary Table S1d). Unique and mappable genes (6 genes) identified from Duroc anti-correlated/Landrace correlated cluster (Table 1) were co-functional with 20 genes based on GeneMania (Table S1b). The gene ontology enrichment analysis of these 26 genes (unique genes from Table 1 and co-functional genes from GeneMania) revealed the ER to Golgi vesicle-mediated transport and membrane fusion (Table S1c) as an enriched biological process. Butanoate metabolism, alanine-aspartate-glutamate metabolism, and valine-leucine-isoleucine degradation were significantly enriched KEGG pathways from the Duroc anti-correlated/Landrace correlated cluster (Supplementary Table S1d). Similarly, from the FE-specific group, unique mapped genes (7 genes) from high-FE correlated/low-FE anti-correlated clusters (Table 2) were co-functional with 20 genes identified from GeneMania (Table S1b). These genes were involved with the cGMP metabolic process, purine nucleotide biosynthesis, and phosphorus-oxygen lyase activity pathways (Table S1c). The top significant KEGG pathway enriched was the cGMP-PKG signaling pathway (Supplementary Table S1d).

## 3. Discussion

FE is an important quantitative trait, which quantifies the efficiency of nutrient conversion from the feed to a tissue that is of nutritional and economic significance [19]. Understanding the molecular mechanisms underlying FE will be advantageous in the efficient selection of pigs and benefit the pig producers. In the Danish pig industry, Duroc is the most popularly used terminal sires in combination with crossbred Landrace X Yorkshire breeds [20], so the selection pressures for FE in Duroc is higher as compared to Landrace. Thus, in the current study, we attempted to identify the gene-metabolite interactions specific to each breed. FE is a complex trait influenced by environmental and health factors and involves many organs. Skeletal muscle, being the largest organ in the body, is an essential location for the metabolism of carbohydrates and lipids [21,22]. It plays a significant role in the utilization and storage of energy acquired from the feed. Thus, understanding the difference in the regulatory processes from a divergent FE group will add a layer of knowledge to the understanding of biological mechanisms involved with this complex trait. 

A plethora of metabolome and transcriptome studies for FE in pigs are reported [3,9,10,12]. However, to the best of our knowledge, markers from the integration of metabolome and transcriptome in Duroc and Landrace pigs has not been done before. Herein, we unveiled the gene-metabolite relationships that are phenotype dependent. This approach highlighted molecular functions and pathways that are strongly evidenced by the integration study. Evaluating phenotype-specific relationships between metabolites and genes assists us to elucidate novel co-regulation patterns that would not be identified by single approaches. In the current study, we integrated untargeted metabolomic and transcriptomic data. We used a numerical data integration approach that employed the integration of a linear model (IntLIM package) to predict metabolite levels from gene-expression in a phenotype dependent manner [23]. 

We attempted to identify the breed-specific and FE-specific gene-metabolite pairs in the current study. The PCA analysis showed a difference in the expression of genes in Duroc and Landrace. However, PCA for the FE group did not exhibit significant clusters between groups, which may be due to the small sample size evaluated here. With our current metabolome-transcriptome analysis, we identified 21 gene-metabolite breed-specific pairs and 12 gene-metabolite FE-specific pairs. 

### 3.1. Breed-Specific Pathway Analysis

**Figure 4:**
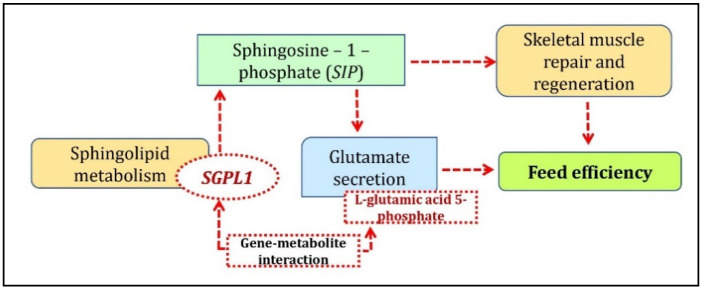
Biological mechanism showing the involvement of *SGPL1*-L-glutamic acid 5-phosphate gene-metabolite pair underlying sphingolipid metabolism identified from Duroc correlated/Landrace anti-correlated cluster.

In the breed-specific analysis, two clusters were identified between Duroc and Landrace breeds. The Duroc correlated/Landrace anti-correlated cluster associated L-glutamic acid 5-phosphate metabolite with the *FAM160A2*, ENSSSCG00000040110, *ETS2*, and *SGPL1* genes; cystathionine ketimine metabolite with ENSSSCG00000040110 and *SGPL1* genes and Rhodamine B with the *SNRPN* genes. The arginine and proline metabolism pathways were associated with the unique metabolites from this cluster. The gene ontology enrichment analysis of the unique genes identified from this cluster with the co-functional genes found enrichment for the multicellular organismal process, sphingolipid catabolic process, regulation of hemostasis, and coagulation pathways. Sphingolipid metabolism associated with the *SGPL1* and *GBA* genes was identified as the top KEGG pathway. *SGPL1* (sphingosine-1-phosphate) catalyzes the final step of the sphingolipid pathway by irreversibly converting sphingosine-1-phosphate (*S1P*) to its by-products [24], thereby regulating *S1P*. *S1P* plays a role as a muscle trophic factor by activating quiescent muscle stem cells (satellite cells) for efficient skeletal muscle repair and regeneration [25]. The role of FE on skeletal muscle mass was well established from previous studies wherein the improvement of muscle properties and an increase in muscle mass is attributed by selection for low RFI in pigs [26]. *S1P* is also reported to trigger glutamate secretion and potentiates depolarization-evoked glutamate secretion [27]. Glutamic acid has been found to play a crucial role in FE as it improves the FE of weaned piglets [28]. This supports the results in the current study where *SGPL1* was associated with L-glutamic acid 5-phosphate as a significant gene-metabolite pair. A brief overview of the role of *SGPL1* in sphingolipid-metabolism regulating FE is given in Figure 4. Therefore, further investigation of *SGPL1*-L-glutamic acid 5-phosphate gene-metabolite pair, which was positively correlated with Duroc while negatively correlated with Landrace concerning FE traits, could be a major avenue for breed-specific research and its effect on FE and meat quality traits.

We also identified the *SNRPN* gene and Rhodamine B relation in the Duroc correlated cluster. A previous study showed the *SNRPN* gene (small nuclear ribonucleoprotein polypeptide N) was ubiquitously expressed in pigs [29]. Small nuclear ribonucleoproteins and heterogeneous small nuclear riboproteins play roles in nucleolar ribosomal RNA (rRNA) and messenger RNA (mRNA) synthesis in conjunction with spliceosome activity responsible for cleaving on introns from the pre mRNA molecule [30]. Furthermore, in a study of FE in broiler chickens, a high FE phenotype exhibited enrichment of ribosome assembly including small nuclear ribonucleoprotein, as well as nuclear transport and protein translation processes than low FE phenotype [31]. 

**Figure 5:**
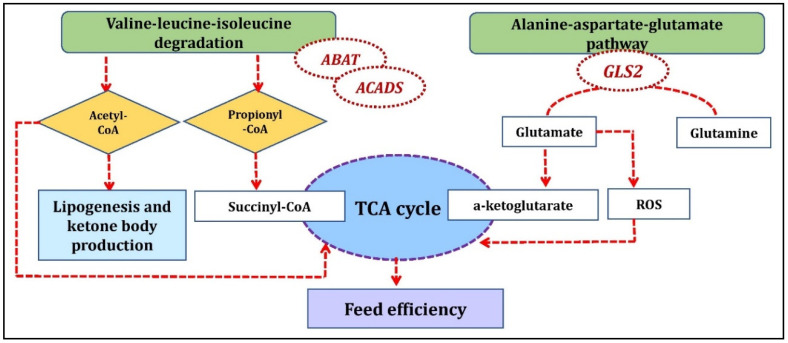
Biological mechanism showing the involvement of genes identified from Duroc anti-correlated/Landrace correlated clusters underlying feed efficiency.

The Duroc anti-correlated/Landrace correlated cluster identified aloesol—*ZC2HC1B*, Proanthocyanidin a2—*ZDHHC22*; fenamiphos metabolite with ENSSSCG00000040467, ENSSSCG00000018649 gene; ganoderenic acid e metabolite with ENSSSCG00000037595 and *SEC22C* genes; *FAM163B* gene with taraxacolide 1-o-b-d-glucopyranoside, theogallin, and ketoleucine metabolites; *LRRTM2* gene with cystathionine ketimine and L-glutamic acid 5-phosphate metabolites and *GLS2* gene with L-glutamic acid 5-phosphate, cystathionine ketimine, and paracetamol sulfate metabolites. One of the significant pathways in Landrace correlated cluster was valine, leucine, and isoleucine degradation which included the *ABAT* and *ACADS* genes (co-functional genes) identified in the current study. Valine, leucine, and isoleucine are branched-chain amino acids (BCAA) and have a crucial role in protein synthesis and energy production [32]. The degradation of BCAA can be glucogenic (valine), ketogenic (leucine and isoleucine), or both (isoleucine). The end products from their degradation, succinyl-CoA and/or acetyl-CoA can enter the tricarboxylic acid (TCA) cycle for energy generation and gluconeogenesis or may act as precursors for lipogenesis and ketone body production through acetyl-CoA and acetoacetate [33]. Glucose metabolism and the TCA pathway in the skeletal muscle is a key pathway regulating FE traits in pigs [34]. In an interesting proteomic study involving glucose metabolism and the TCA cycle reported earlier, the proteins catalyzing the conversion of glucose to pyruvate and oxaloacetate were up-regulated in high-FE pigs while those involved in the conversion of pyruvate to lactate or acetyl-CoA were down-regulated in high-FE pigs [34]. This resulted in inhibition of the TCA cycle in high-FE pigs due to the down-regulation of key catalytic proteins [34]. Thus, the pathway identified with BCAA in the current study may cause differences in FE concerning specific breed as evident from the indirect link with TCA and FE (Figure 5). The BCAA also affects protein synthesis, as reported earlier in a study with reduced degradation of rat skeletal muscle proteins [35]. Additionally, leucine activates mTOR signaling, one of the central regulators of cell growth and metabolism along with an increase in fatty acid oxidation. With all these supporting facts of BCAA regulating cellular metabolism, protein, and fatty acid degradation, which are also key factors influencing FE, the role of the valine-leucine-isoleucine pathway in FE cannot be overlooked. Furthermore, this pathway has been found over-represented in a GWAS study for RFI in beef cattle [36] and pigs [3].

We identified the alanine-aspartate-glutamate metabolism KEGG pathway involving the *GLS2* and *ABAT* genes within the Duroc anti-correlated/Landrace correlated cluster. *GLS2* is a mitochondrial glutaminase that catalyzes glutamine to glutamate which is further converted to a-ketoglutarate, an important substrate for the citric acid cycle to produce ATP in mitochondria. Glutamate is also a precursor of reduced glutathione (*GSH*), an important antioxidant molecule, and a scavenger for ROS (Reactive oxygen species) [37]. ROS are regulated by FE related traits as reported from the previous studies, higher levels of ROS production and oxidized mitochondrial proteins have been found in the muscle of low FE chickens [38] while in pigs, ROS production in mitochondria was higher in semitendinosus muscle of less efficient pigs selected for high RFI compared to high efficient pigs (low RFI) [39]. *GLS2* interacted with L-glutamic acid 5-phosphate and cystathionine ketimine metabolites as identified from Duroc anti-correlated/Landrace correlated clusters (Figure 5). 

The unique metabolites identified in the breed-specific clusters were also previously reported in another study for the metabolomic analysis of FE related traits in Duroc and Landrace [3]. The breed-specific unique metabolites such as aloesol and ketoleucine affected FE in Duroc [3]. In contrast, rhodamine B, taraxacolide 1-o-b-d-glucopyranoside, and ganoderenic acid were underlying testing daily gain (TDG) in Duroc [3]. Theogallin and ketoleucine were involved with TDG and daily gain (DG) in Duroc and Landrace and RFI in Duroc [3]. L-glutamic acid 5-phosphate, cystathionine ketimine, and paracetamol sulfate were associated with FE and RFI in Landrace [3]. It is worth highlighting the interaction of metabolites L-glutamic acid 5-phosphate and cystathionine ketimine identified in this study. While these metabolites interact with the *SGPL1* gene as identified in the Duroc correlated cluster, on the contrary, they interact with the *GLS2* gene in the Landrace correlated cluster. Both *SGPL1* and *GLS2* were in the top significant pathways in their respective cluster. Therefore, these gene-metabolite interactions which are highly specific to breed differences open up the avenues for further research to extrapolate differences in FE related traits concerning breeds.

### 3.2. cGMP-PKG Pathway Involved with FE-Specific Analysis

**Figure 6:**
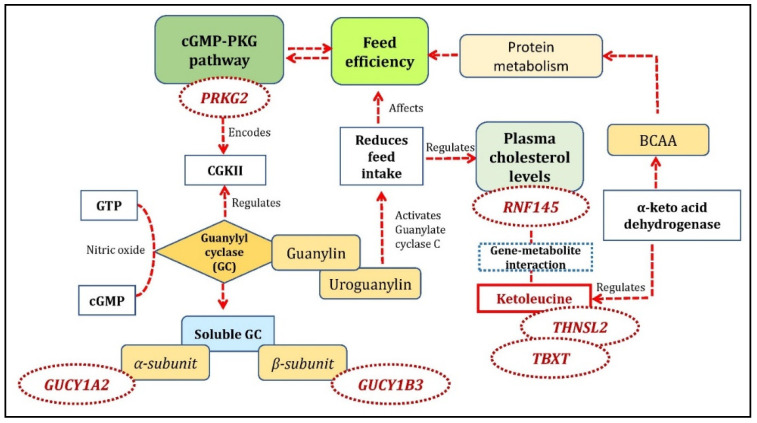
An overview of gene-metabolite pairs affecting the cGMP-PKG pathway involved with feed efficiency.

In the FE-specific analysis, we found 12 significant gene-metabolite pairs. The gene-metabolite pairs in high-FE correlated/low-FE anti-correlated cluster were *TBXT* gene with theogallin and ketoleucine metabolites; *THNSL2* gene with pyrocatechol, 2-pyrocatechuic acid, ketoleucine, and theogallin metabolites; *TUBAL3* gene with neodispyrin metabolite, *RNF145* gene with ketoleucine metabolite, *ENAM* gene and U2 snRNA with proanthocyanidin a2 metabolite, ENSSSCG00000038441 gene with adrenochrome metabolite, and *PRKG2* gene with levulinic acid metabolite. The pathway analysis with the unique metabolites identified from high-FE correlated/low-FE anti-correlated clusters was over-represented for valine-leucine-isoleucine biosynthesis and degradation pathway. The unique mapped genes and co-functional genes were enriched for the following biological processes: lyase activity, cGMP metabolic process, phosphorus-oxygen lyase activity, and cyclic purine nucleotide metabolic process. cGMP-PKG, purine metabolism, and renin secretion were the KEGG pathways identified in this cluster. The cGMP pathways were also identified in the studies reported earlier with FE related traits with pigs [40] and beef cattle [41]. The *PRKG2* gene, one of the main predictors for cGMP pathways and also identified in this study, encodes the serine/threonine-protein, which binds to inhibits the activation of several receptor tyrosine kinases and is a regulator of the intestinal secretion, bone growth, and renin secretion (https://www.ncbi.nlm.nih.gov/gene/5593). *PRKG2* encodes for CGKII (guanosine 3,5-cyclic monophosphate (cGMP)-dependent protein kinase II) and is abundantly expressed in intestinal epithelium. *CGKII* relays signaling through a membrane-associated, cGMP-producing enzyme, guanylyl cyclase (GC). The catalytic activity of this receptor-enzyme is triggered by two locally produced ligands, the peptides guanylin and uroguanylin [42]. The GC is activated by nitric oxide (NO) and catalyzes the conversion of intracellular guanosine-5′-triphosphate (GTP) to cyclic guanosine-3’,5’-monophosphate (cGMP). This enzyme has two forms: a membrane protein and a soluble form with specific kinetic properties and tissue distributions. The soluble GC (sGC) form is a heterodimeric protein consisting of α (α_1_ and α_2_,) and β (β_1_ and β_2_) subunits encoded by distinct genes [43]. An alpha subunit of guanylyl cyclase, *GUCY1A2* and a beta subunit *GUCY1B3* was identified as co-functional genes in the current study and were involved with cGMP, phosphorus metabolic process, nitrogen metabolic process pathways as identified in the current study. Uroguanylin is a gastrointestinal hormone primarily involved in fluid and electrolyte handling. It has recently been reported that prouroguanylin, secreted postprandially, is converted to uroguanylin in the brain and activates the receptor guanylate cyclase-C (GC-C) to reduce food intake in mice [44]. Reduced FI is a characteristic feature for the selection of the pigs known for high FE [1]. The overview of the mechanism involving the cGMP-PKG pathway and its role in FE is given in Figure 6.

A positive correlation between FI and plasma cholesterol levels is previously established in pigs [45]. *RNF145*, which was positively correlated in the high-FE cluster participates in key signaling pathways of cholesterol homeostasis [46]. *RNF145* expression is induced in response to LXR activation and high-cholesterol diet feeding [46]. Transduction of *RNF145* into mouse liver inhibits the expression of genes involved in cholesterol biosynthesis and reduces plasma cholesterol levels. On the other hand, its inactivation increases cholesterol levels both in the liver and plasma [46]. In this study, *RNF145* was identified with ketoleucine as a significant gene-metabolite pair in this cluster (Figure 6). Ketoleucine is an abnormal metabolite that arises from the incomplete breakdown of branched-chain amino acids (https://hmdb.ca/metabolites/HMDB0000695). Ketoleucine is regulated by branched-chain α-keto acid dehydrogenase. Studies reported that the branched-chain α-keto acid dehydrogenase catalyzes the irreversible oxidative decarboxylation of all three branched-chain keto acids (BCKA) derived from branched-chain amino acids (BCAA), i.e., α-ketoisocaproate (ketoleucine) [47]. They demonstrated changes in BCKA activity that showed a significant alteration in BCAA and protein metabolism during starvation in rats [47]. BCAA also plays a crucial role in FE by regulating energy homeostasis in addition to lipid and protein metabolism as reported in pigs [48].

Apart from *RNF145*, gene-metabolite interaction of ketoleucine with *TBXT* and *THNSL2* was also identified in this study. *THNSL2* was reported among the top 40 significantly differentially expressed genes of characterized proteins between high- and low-ADG steers from a liver transcriptome profiling of beef cattle [49]. This gene has also been associated with abdominal and visceral fat in humans based on GWAS [50]. An interaction of proanthocyanidin a2 with ENSSSCG00000019329 (U2 snRNA) was also identified with low but positive correlation with high-FE cluster while a negative correlation with the low-FE cluster. U2 spliceosomal snRNAs are the molecules found in the major spliceosomal machinery of all eukaryotic organisms and affect their gene expression [51]. U2 snRNA plays a central role in the splicing of mRNA precursors by regulating a dynamic set of RNA-RNA base-pairing interactions [52]. From the previously reported studies, the role of precursor mRNA in gene expression has been established as it removes the intronic sequence from immature RNA, leading to a production of mature mRNA that might differ in function [53]. Regulation of pre-mRNA splicing by nutrients modulates the carbohydrate and lipid metabolism [53]. U2 interacts with proanthocyanidin a2 in the current study. Proanthocyanidin a2 is an antioxidant and has a broad spectrum of biologic properties against oxidative stress [54]. Proanthocyanidin significantly increased the activity of antioxidant enzymes such as superoxide dismutase, glutathione peroxidase, and catalase [54]. The role of antioxidant activity with FE was reported earlier in beef cattle as low feed efficient steers had greater superoxide dismutase and glutathione peroxidase activity than the high feed efficient ones, potentially using a greater proportion of energy [55]. Thus, as evident from all these facts, the potential role of proanthocyanidin a2–U2 interaction in high-FE pigs identified in the current study might be interesting to explore.

The gene-metabolite pairs identified in the present study over-represented some pathways that have been reported to have a role in FE related traits. Some of the genes identified are novel and were not included in the pathway analysis. Since these gene-metabolite pairs selected have a highly significant correlation with respect to each study group, a detailed study of these genes and metabolites are needed to better understand their role in FE related-traits. Further studies on the identified gene-metabolite pairs may assist in the discovery of biomarkers as these significant pairs identified directly reflect the phenotype as revealed by the candidate gene-expression with the downstream metabolite differences in pigs with low and high FE groups.

## 4. Materials and Methods

### 4.1. Data Resource and Phenotype Generation

The pigs used in this experiment were raised at the pig testing station “Bøgildgård” operated by SEGES within Landbrug and Fødevarer (L&F: Danish Agriculture and Food Council). Pigs were *ad libitum* fed and free water supplied. The authors of this study were not responsible for animal husbandry, diet, and care as the testing station is a facility within the Danish breeding program run by SEGES. The initial bodyweight of the pigs before the testing period was approximately 7 kg, followed by a 5-week acclimatization phase. For the phenotypic traits, we calculated FCR and RFI, as reported in our previous study [40]. We considered the same classification of animals in this study as efficient and inefficient (low and high FCR, respectively), as previously reported [40]. The classification was done by selecting pigs that were one standard deviation above or below the mean FCR for each breed as previously reported. 

### 4.2. Gene Expression Profile, Metabolite Profile, and Data Analyses

For transcriptome analysis, we collected *psoas major* muscle from 40 purebred uncastrated male pigs from two breeds comprising of 12 Danbred Duroc and 28 Danbred Landrace. The tissue samples were preserved in RNAlater (Ambion, Austin, TX, USA) immediately post-slaughter and stored at −25 °C until subsequent analysis. Total RNA isolation and sequencing were carried out by BGI Genomics. Paired-end sequencing (100 bp) was performed on the BGISEQ-500 platform after Oligo dT library preparation. Read quality control, mapping, and gene counts were reported elsewhere [14]. Lowly expressed genes were filtered out, and the gene counts normalization was carried out by applying the variance stabilizing transformation (*VST*) function from DeSeq2 [56]. 

To identify significant gene-metabolite pairs, we analyzed the data considering two approaches, i.e., breed-specific (Duroc-Landrace) and FE-specific (low-high FE groups). Thus, we fitted a linear model for adjusting the read counts with the covariates using the Limma R package [57]. For adjusting the data to identify breed-specific differences, we adopted the following model:

$$y_{ijkl}=\mu +F_{i}+R_{j}+A_{k}+P_{l}+\varepsilon_{ijkl}$$

where $y_{ijkl}$: is the normalized read counts; µ: is the intercept; $F_{i}$: is the fixed effect of the FE group (two levels, high and low); $R_{j}$: is the covariate for the RIN values; $A_{k}$: is the covariate for the animal’s slaughter age, in days; $P_{l}$: is the fixed effect of the pen (8 levels); $\varepsilon_{ijkl}$: is the random residual effect associated with each observation.

To identify differences between high and low FE groups, the breed effect was added in the linear model, as follows:

$$y_{ijkl}=\mu +B_{i}+R_{j}+A_{k}+P_{l}+\varepsilon_{ijkl}$$

where $y_{ijkl}$: is the normalized read counts; µ: is the intercept; $B_{i}$: is the fixed effect of the breed (two levels, Duroc and Landrace); $R_{j}$: is the covariate for the RIN values; $A_{k}$: is the covariate for the animal’s slaughter age, in days; $P_{l}$: is the fixed effect of the pen (eight levels); $\varepsilon_{ijkl}$: is the random residual effect associated with each observation.

Regarding the identification of the metabolites, we used an untargeted metabolomic approach, as reported elsewhere [3]. In summary, 5 mL of blood samples at two-time points were collected from jugular venipuncture of each non-fasted pig into the EDTA tubes and immediately placed on ice. The plasma samples extracted from 109 animals (59 Duroc and 50 Landrace) were subjected to metabolomics analysis, as described in a previous study [3]. The metabolite data from this study were accessed using MetaboLights accession ID MTBLS1384 with a link: https://www.ebi.ac.uk/metabolights/MTBLS1384. Due to the need for paired data to carry the integrative analysis, only those samples with both metabolite and RNA-Seq data were used herein. The metabolite data from time-point two in 40 pigs were log-normalized before fitting into a linear model. Only those with the relative standard deviation > 0.15 were used based on the raw counts. As proposed for the RNA-seq, we adjusted the log-normalized metabolite data considering both approaches. First, the following model was employed for the breed-specific analysis:

$$m_{ijkl}=\mu +F_{i}+D_{j}+A_{k}+P_{l}+\varepsilon_{ijkl}$$

where $m_{ijkl}$: is the is the log-normalized concentration of each metabolite (*n* = 749); µ: is the intercept; $F_{i}$: is the fixed effect of the FE group (two levels, high and low); $D_{j}$: is the fixed effect of the batch for metabolomic analysis (two levels); $A_{k}$: is the covariate for the sampling age, in days; $P_{l}$: is the fixed effect of the pen (8 levels); $\varepsilon_{ijkl}$: is the random residual effect associated with each observation.

For the FE-specific group approach, we fitted the data as follows:

$$m_{ijkl}=\mu +B_{i}+D_{j}+A_{k}+P_{l}+\varepsilon_{ijkl}$$

where $m_{ijkl}$: is the is the log-normalized concentration of each metabolite (*n* = 749); µ: is the intercept; $B_{i}$: is the fixed effect of the breed (two levels, Duroc and Landrace); $D_{j}$: is the fixed effect of the batch for metabolomic analysis (two levels); $A_{k}$: is the covariate for the sampling age, in days; $P_{l}$: is the fixed effect of the pen (8 levels); $\varepsilon_{ijkl}$: is the random residual effect associated with each observation.

The metabolites were annotated with HMDB (Human metabolome database) based on library search of the masses in HMDB with a mass uncertainty of 0.005 Da or 5 ppm. Those metabolites that did not correspond to HMDB entries were left unannotated and removed from the analysis.

### 4.3. Integration of Transcriptomic and Metabolomic Data Based on the Linear Model

To uncover the complex relationship between metabolites and genes, we adopted a linear model framework using the IntLIM (Integration of Linear model) R-package (version 0.1.0) (https://github.com/mathelab/IntLIM) [23]. The IntLIM approach allows us to integrate the metabolomic-transcriptomic data considering a case-control design. Thus, as initially proposed, we compared the breeds (Duroc vs. Landrace) and the FE groups (low and high FCR animals). As a quality control step from IntLIM, we filtered out genes with the lowest 5% of the variation. Gene and metabolite exploratory analyses were performed by applying Principal Component Analysis to identify breed- and FE-specific clusters. 

The linear model for data integration is given as described in the following equation:

$$m=\beta_{1}+\beta_{2}g+\beta_{3}p+\beta_{4}\left(g:p\right)+\varepsilon$$

where m: is the log-normalized metabolite abundance; $\beta_{1}:$ is the intercept; $\beta_{2}g$: is the normalized and adjusted gene expression level; $\beta_{3}p$: is the phenotype (FE group—high and low FE; or breed—Duroc and Landrace); $\beta_{4}\left(g:p\right):\;$is the interaction between gene expression and phenotype; ε: is the residual effect associated with each observation (ε  =  N(0, σ)).

A statistically significant two-tailed *p*-value of the gene-phenotype (g-p) interaction indicates the difference in the phenotype of FE groups (high and low) or breed (Duroc and Landrace) calculated by the slope relating gene-expression and metabolite abundance [23]. The two-tailed *p*-value indicates that the slope relating gene-expression and metabolite abundance is different from one phenotype compared to the other. Thus, it was used to identify gene-metabolite associations that are specific to a particular phenotype (breed—Duroc and Landrace, FE—low and high). We calculated the absolute difference in the Spearman correlation identified from IntLIM between the FE and breed groups to find the significant (*p* < 10^−7^) gene-metabolite pairs. The absolute difference between the FE group was estimated as ($r_{Low\_cor}-r_{High\_cor}$) where ($r_{High\_cor})\;\mathrm{is}$ the correlation given for a gene-metabolite pair in high-FE group (Table 2) while $(r_{Low\_cor}$) is the correlation given for a gene-metabolite pair in the low-FE group (Table 2). The absolute difference in the correlation between the breeds was estimated as ($r_{Landrace\_cor}-r_{Duroc\_cor}$), where, ($r_{Duroc\_cor})$ is the correlation for a given gene-metabolite pair in Duroc (Table 1) while $(r_{Landrace\_cor}$) is the correlation for a gene-metabolite pair in Landrace (Table 1). 

### 4.4. Pathway Over-Representation Analysis

In the breed-specific and FE-specific group, the same metabolite can be related to more than one gene and vice-versa. So, we screened for the common metabolites and genes in the group and referred them as unique metabolites and unique genes respectively in this study. We analyzed the unique metabolites in each group (breed-specific and FE-specific) using Metaboanalyst 4.0 (www.metaboanalyst.ca) [58]. We used three parameters for the pathway analysis: the pathway library, algorithm for pathway over-representation analysis, and algorithm for topological analysis. For the current study, we selected the *Homo sapiens* (KEGG) pathway library to estimate the importance of the compound in a given metabolic pathway. For pathway over-representation and topology analysis, we used the hypergeometric test and relative-betweenness centrality algorithm, respectively, to measure the connections with the other nodes, including the number of shortest paths going through the node of interest. 

Regarding the unique mapped (with chromosomal location information) genes, we carried out a co-functionality analysis using GeneMANIA [59] (www.genemania.org). GeneMANIA considers our query list of unique genes identified in each cluster (breed-specific and FE-specific) and allows us to predict the co-functional genes underlying similar functions. Thus, we analyzed the unique genes in each cluster (breed-specific—Duroc correlated cluster, and Duroc anti-correlated cluster, FE-specific—High-FE correlated cluster) to identify the co-functional genes in each cluster. Next, we used the unique genes, as well as the co-functional genes in each cluster of each group, to identify the GO terms using GOrilla (Gene ontology enrichment analysis and visualization tool) (http://cbl-gorilla.cs.technion.ac.il/) [60]. To this end, the *Homo sapiens* were used as the reference, and the entire set of identified and annotated genes in this study (*n* = 15,187 genes) was used as a background. Over-representation KEGG pathway analysis with the unique and co-functional genes in each cluster was performed using ClueGO version 2.5.4 [61] to cluster redundant terms with a kappa score of 0.4 and *S. scrofa* annotation as the background. The pathways were selected after filtering for group *p*-value corrected with Bonferroni step down ≤ 0.05 and those with at-least two over-represented genes. 

## 5. Conclusions

This study applied a novel approach for metabolome-transcriptome data integration using the linear model unveiling potential gene-metabolite pairs affecting the biological processes related to FE in pigs. To the best of our knowledge, this is the first study to report the gene-metabolite interaction mechanisms that may determine nutrient partitioning and energy utilization and hence affect FE in pigs. The approach followed here provided many interesting genes and metabolites with significant *p*-values. While some of the metabolites and genes identified were known with their association for FE, others are novel and provide new avenues for further research. The unique metabolites were associated with valine-leucine-isoleucine biosynthesis/degradation and arginine-proline metabolism. The unique genes enriched for sphingolipid metabolism, valine-leucine-isoleucine degradation, alanine-aspartate-glutamate pathway (breed-specific), and cGMP-PKG signaling pathway (FE-specific). Further validation of genes, metabolites, and gene-metabolite interactions in a cohort with more animals with additional features such as alteration in dietary components, farm variations, and other environmental effects would help to establish a framework for future FE prediction using metabolomics biomarker profiles that could be practical to use in large populations other than genomic profiling. More data would also make it possible to model the complex relations in gene-metabolite profiles over time more accurately and will help to elucidate the regulatory mechanisms affecting the pathways underlying FE.

## Figures and Tables

**Table 1:** Gene-metabolite interaction pairs from IntLIM for the breed-specific groups.

Metabolite_Name	Ensembl ID	Gene Name	Duroc_cor	Landrace_cor	Abs diff.corr	Pval	FDRadjPval
Rhodamine B	ENSSSCG00000028124	*SNRPN*	0.776224	−0.54242	−1.31864	3.11 × 10^−7^	0.1
L-glutamic acid 5-phosphate	ENSSSCG00000010274	*SGPL1*	0.888112	−0.21456	−1.10267	1.57 × 10^−7^	0.09
Cystathionine ketimine	ENSSSCG00000010274	*SGPL1*	0.874126	−0.18719	−1.06132	2.67 × 10^−7^	0.1
Cystathionine ketimine	ENSSSCG00000040110	*Novel_gene*	0.923077	−0.11385	−1.03692	1.08 × 10^−8^	0.02
L-glutamic acid 5-phosphate	ENSSSCG00000038948	*ETS2*	0.895105	−0.11002	−1.00512	2.66 × 10^−8^	0.02
L-glutamic acid 5-phosphate	ENSSSCG00000040110	*Novel_gene*	0.937063	0.010947	−0.92612	1.48 × 10^−9^	0.01
L-glutamic acid 5-phosphate	ENSSSCG00000014632	*FAM160A2*	0.951049	0.229885	−0.72116	1.09 × 10^−7^	0.08
Aloesol	ENSSSCG00000004128	*ZC2HC1B*	−0.22767	0.048714	0.276385	4.33 × 10^−7^	0.1
Theogallin	ENSSSCG00000026442	*FAM163B*	−0.29772	0.45156	0.749284	1.69 × 10^−7^	0.09
Fenamiphos	ENSSSCG00000018649	*Novel_gene*	−0.68652	0.093596	0.780112	3.44 × 10^−7^	0.1
Taraxacolide 1-o-b-d-glucopyranoside	ENSSSCG00000026442	*FAM163B*	−0.27321	0.648057	0.921262	1.55 × 10^−7^	0.09
Proanthocyanidin a2	ENSSSCG00000033688	*ZDHHC22*	−0.63047	0.32567	0.956144	4.20 × 10^−7^	0.1
Fenamiphos	ENSSSCG00000040467	*Novel_gene*	−0.67251	0.288998	0.961504	2.25 × 10^−8^	0.02
L-glutamic acid 5-phosphate	ENSSSCG00000000401	*GLS2*	−0.85315	0.109469	0.962616	1.80 × 10^−7^	0.09
Paracetamol sulfate	ENSSSCG00000000401	*GLS2*	−0.94406	0.038314	0.98237	2.87 × 10^−7^	0.1
L-glutamic acid 5-phosphate	ENSSSCG00000034989	*LRRTM2*	−0.83916	0.15052	0.989681	2.17 × 10^−8^	0.02
Cystathionine ketimine	ENSSSCG00000034989	*LRRTM2*	−0.79021	0.204707	0.994917	2.53 × 10^−8^	0.02
Ketoleucine	ENSSSCG00000026442	*FAM163B*	−0.5289	0.466886	0.995783	1.19 × 10^−8^	0.02
Ganoderenic acid e	ENSSSCG00000034200	*SEC22C*	−0.85315	0.288998	1.142145	3.47 × 10^−7^	0.1
Cystathionine ketimine	ENSSSCG00000000401	*GLS2*	−0.93007	0.249042	1.179112	2.92 × 10^−9^	0.01
Ganoderenic acid e	ENSSSCG00000037595	*Novel_gene*	−0.85315	0.449371	1.302517	2.26 × 10^−7^	0.1

Duroc_cor: Spearman correlation in Duroc; Landrace_cor: Spearman correlation in Landrace; Abs diff.corr: the absolute difference in correlation between Duroc and Landrace; Pval: *p*-value (*p* < 10^−7^ was selected as the cut-off value); FDRadjPval: FDR adjusted *p*-value.

**Table 2:** Gene-metabolite interaction pairs from IntLIM for the FE-specific groups.

Metabolite_Name	Ensembl ID	Gene Name	High_cor	Low_cor	Abs diff.corr	Pval	FDRadjPval
Pyrocatechol	ENSSSCG00000025106	*THNSL2*	0.66996	−0.73284	−1.4028	4.72 × 10^−8^	0.06
2-pyrocatechuic acid	ENSSSCG00000025106	*THNSL2*	0.645257	−0.68137	−1.32663	7.13 × 10^−8^	0.08
Ketoleucine	ENSSSCG00000017043	*RNF145*	0.548419	−0.75735	−1.30577	2.17 × 10^−7^	0.1
Ketoleucine	ENSSSCG00000025106	*THNSL2*	0.498024	−0.58088	−1.07891	1.19 × 10^−7^	0.10
Theogallin	ENSSSCG00000036609	*TBXT*	0.727273	−0.35049	−1.07776	2.87 × 10^−8^	0.05
Neodiospyrin	ENSSSCG00000029077	*TUBAL3*	0.608696	−0.46814	−1.07683	2.63 × 10^−8^	0.05
Theogallin	ENSSSCG00000025106	*THNSL2*	0.4417	−0.6152	−1.0569	2.52 × 10^−7^	0.1
Proanthocyanidin a2	ENSSSCG00000008938	*ENAM*	0.37954	−0.60539	−0.98493	1.99 × 10^−7^	0.1
Ketoleucine	ENSSSCG00000036609	*TBXT*	0.557312	−0.3701	−0.92741	8.78 × 10^−8^	0.08
Adrenochrome	ENSSSCG00000038441	*Novel_gene*	0.171485	−0.58088	−0.75237	1.71 × 10^−8^	0.05
Proanthocyanidin a2	ENSSSCG00000019329	*U2*	0.052384	−0.66176	−0.71415	1.11 × 10^−8^	0.05
Levulinic acid	ENSSSCG00000009250	*PRKG2*	0.075117	−0.54902	−0.62414	1.93 × 10^−7^	0.1

High_cor: Spearman correlation in high FE group; Low_cor: Spearman correlation in low FE group; Abs diff.corr: the absolute difference in correlation between the high and low FE group; Pval: *p*-value (*p* < 10^−7^ was selected as the cut-off value); FDRadjPval: FDR adjusted *p*-value.

## Data Availability

The metabolites dataset generated and analyzed during the current study is publicly available at the Metabolights database https://www.ebi.ac.uk/metabolights/MTBLS1384 with accession ID: MTBLS1384. (https://doi.org/10.1093/nar/gks1004; PubMed PMID: 2310955). The transcriptome dataset analyzed during the present study is also publicly available at the NCBI-GEO database with Accession ID GSE148889 and can be accessed at https://www.ncbi.nlm.nih.gov/geo/query/acc.cgi?acc=GSE148889.

## Supplementary Materials

The following are available online at https://www.mdpi.com/2218-1989/10/7/275/s1, Figure S1: The principal component analysis of metabolites and genes. A and B: PCA plot of genes and metabolites, respectively, in (1) Duroc and (2) Landrace; C and D: PCA plot of genes and metabolites, respectively, in high and low feed efficient groups, Table S1: Pathways enriched by unique gene-metabolite pairs. Spreadsheet tabs are divided into (a) metabolite enrichment analysis results by Metaboanalyst; (b) list of the co-functional genes by GeneMania; (c) gene ontology pathways enriched by Gorilla; (d) KEGG pathways enriched by ClueGO.

## References

[1] Patience J.F., Rossoni-Serão M.C., Gutiérrez N.A. (2015). A review of feed efficiency in swine: biology and application. J. Anim. Sci. Biotechnol..

[2] He B., Li T., Wang W., Gao H., Bai Y., Zhang S., Zang J., Li D., Wang J. (2019). Metabolic characteristics and nutrient utilization in high-feed-efficiency pigs selected using different feed conversion ratio models. Sci. China Life Sci..

[3] Carmelo V.A.O., Banerjee P., da Silva Diniz W.J., Kadarmideen H.N. (2020). Metabolomic networks and pathways associated with feed efficiency and related-traits in Duroc and Landrace pigs. Sci. Rep..

[4] Godinho R.M., Bergsma R., Silva F.F., Sevillano C.A., Knol E.F., Lopes M.S., Lopes P.S., Bastiaansen J.W.M., Guimarães S.E.F. (2018). Genetic correlations between feed efficiency traits, and growth performance and carcass traits in purebred and crossbred pigs. J. Anim. Sci..

[5] Ren P., Yang X.J., Cui S.Q., Kim J.S., Menon D., Baidoo S.K. (2017). Effects of different feeding levels during three short periods of gestation on gilt and litter performance, nutrient digestibility, and energy homeostasis in gilts. J. Anim. Sci..

[6] Do D.N., Strathe A.B., Ostersen T., Pant S.D., Kadarmideen H.N. (2014). Genome-wide association and pathway analysis of feed efficiency in pigs reveal candidate genes and pathways for residual feed intake. Front. Genet..

[7] Novais F.J., Dromms R.A., Alexandre P.A., Pires P.R.L., Styczynski M.P.-W., Ferraz J.B.S., Fukumasu H., Iglesias A.H. (2019). Identification of a metabolomic signature associated with feed efficiency in beef cattle. BMC Genom..

[8] Rohart F., Paris A., Laurent B., Canlet C., Molina J., Mercat M.J., Tribout T., Muller N., Iannuccelli N., Villa-Vialaneix N. (2012). Phenotypic prediction based on metabolomic data for growing pigs from three main european breeds. J. Anim. Sci..

[9] D’Alessandro A., Marrocco C., Zolla V., D’Andrea M., Zolla L. (2011). Meat quality of the longissimus lumborum muscle of Casertana and Large White pigs: Metabolomics and proteomics intertwined. J. Proteom..

[10] Bertram H.C., Oksbjerg N., Young J.F. (2010). NMR-based metabonomics reveals relationship between pre-slaughter exercise stress, the plasma metabolite profile at time of slaughter, and water-holding capacity in pigs. Meat Sci..

[11] Jing L., Hou Y., Wu H., Miao Y., Li X., Cao J., Michael Brameld J., Parr T., Zhao S. (2015). Transcriptome analysis of mRNA and miRNA in skeletal muscle indicates an important network for differential Residual Feed Intake in pigs. Sci. Rep..

[12] Vincent A., Louveau I., Gondret F., Tréfeu C., Gilbert H., Lefaucheur L. (2015). Divergent selection for residual feed intake affects the transcriptomic and proteomic profiles of pig skeletal muscle. J. Anim. Sci..

[13] Horodyska J., Hamill R.M., Reyer H., Trakooljul N., Lawlor P.G., Mccormack U.M., Wimmers K. (2019). RNA-Seq of Liver From Pigs Divergent in Feed Efficiency Highlights Shifts in Macronutrient Metabolism, Hepatic Growth and Immune Response. Front. Genet..

[14] Carmelo V.A.O., Kadarmideen H.N. (2020). Genome Regulation and Gene Interaction Networks Inferred From Muscle Transcriptome Underlying Feed Efficiency in Pigs. Front. Genet..

[15] Kadarmideen H.N. (2014). Genomics to systems biology in animal and veterinary sciences: Progress, lessons and opportunities. Livest. Sci..

[16] Suravajhala P., Kogelman L.J.A., Kadarmideen H.N. (2016). Multi-omic data integration and analysis using systems genomics approaches: methods and applications in animal production, health and welfare. Genet. Sel. Evol..

[17] Carrillo J.A., He Y., Li Y., Liu J., Erdman R.A., Sonstegard T.S., Song J. (2016). Integrated metabolomic and transcriptome analyses reveal finishing forage affects metabolic pathways related to beef quality and animal welfare. Sci. Rep..

[18] Cavill R., Jennen D., Kleinjans J., Briedé J.J. (2016). Transcriptomic and metabolomic data integration. Brief. Bioinform..

[19] Wilkinson J.M. (2011). Re-defining efficiency of feed use by livestock. Animal.

[20] Do D.N., Strathe A.B., Jensen J., Mark T., Kadarmideen H.N. (2013). Genetic parameters for different measures of feed efficiency and related traits in boars of three pig breeds. J. Anim. Sci..

[21] Morales P.E., Bucarey J.L., Espinosa A. (2017). Muscle Lipid Metabolism: Role of Lipid Droplets and Perilipins. J. Diabetes Res..

[22] Pedersen B.K. (2013). Muscle as a Secretory Organ. Comprehensive Physiology.

[23] Siddiqui J.K., Baskin E., Liu M., Cantemir-Stone C.Z., Zhang B., Bonneville R., McElroy J.P., Coombes K.R., Mathé E.A. (2018). IntLIM: integration using linear models of metabolomics and gene expression data. BMC Bioinform..

[24] Taylor V.A., Stone H.K., Schuh M.P., Zhao X., Setchell K.D., Erkan E. (2019). Disarranged Sphingolipid Metabolism From Sphingosine-1-Phosphate Lyase Deficiency Leads to Congenital Nephrotic Syndrome. Kidney Int. Rep..

[25] Donati C., Cencetti F., Bruni P. (2013). Sphingosine 1-phosphate axis: a new leader actor in skeletal muscle biology. Front. Physiol..

[26] Lefaucheur L., Lebret B., Ecolan P., Louveau I., Damon M., Prunier A., Billon Y., Sellier P., Gilbert H. (2011). Muscle characteristics and meat quality traits are affected by divergent selection on residual feed intake in pigs1. J. Anim. Sci..

[27] Kajimoto T., Okada T., Yu H., Goparaju S.K., Jahangeer S., Nakamura S. (2007). Involvement of Sphingosine-1-Phosphate in Glutamate Secretion in Hippocampal Neurons. Mol. Cell. Biol..

[28] Santos L.S., Miassi G.M., Tse M.L.P., Gomes L.M., Berto P.N., Denadai J.C., Caldara F.R., Dalto D.B., Berto D.A. (2019). Growth performance and intestinal replacement time of 13C in newly weaned piglets supplemented with nucleotides or glutamic acid. Livest. Sci..

[29] Wang M., Zhang X., Kang L., Jiang C., Jiang Y. (2012). Molecular characterization of porcine NECD, SNRPN and UBE3A genes and imprinting status in the skeletal muscle of neonate pigs. Mol. Biol. Rep..

[30] Wahl M.C., Will C.L., Lührmann R. (2009). The Spliceosome: Design Principles of a Dynamic RNP Machine. Cell.

[31] Bottje W.G., Lassiter K., Piekarski-Welsher A., Dridi S., Reverter A., Hudson N.J., Kong B.-W. (2017). Proteogenomics Reveals Enriched Ribosome Assembly and Protein Translation in Pectoralis major of High Feed Efficiency Pedigree Broiler Males. Front. Physiol..

[32] Zhang S., Zeng X., Ren M., Mao X., Qiao S. (2017). Novel metabolic and physiological functions of branched chain amino acids: a review. J. Anim. Sci. Biotechnol..

[33] Harper A.E., Miller R.H., Block K.P. (1984). Branched-chain amino acid metabolism. Annu. Rev. Nutr..

[34] Fu L., Xu Y., Hou Y., Qi X., Zhou L., Liu H., Luan Y., Jing L., Miao Y., Zhao S. (2017). Proteomic analysis indicates that mitochondrial energy metabolism in skeletal muscle tissue is negatively correlated with feed efficiency in pigs. Sci. Rep..

[35] Freund H.R., Hanani M. (2002). The metabolic role of branched-chain amino acids. Nutrition.

[36] Duarte D.A.S., Newbold C.J., Detmann E., Silva F.F., Freitas P.H.F., Veroneze R., Duarte M.S. (2019). Genome-wide association studies pathway-based meta-analysis for residual feed intake in beef cattle. Anim. Genet..

[37] Hu W., Zhang C., Wu R., Sun Y., Levine A., Feng Z. (2010). Glutaminase 2, a novel p53 target gene regulating energy metabolism and antioxidant function. Proc. Natl. Acad. Sci. USA.

[38] Zampiga M., Flees J., Meluzzi A., Dridi S., Sirri F. (2018). Application of omics technologies for a deeper insight into quali-quantitative production traits in broiler chickens: A review. J. Anim. Sci. Biotechnol..

[39] Grubbs J.K., Fritchen A.N., Huff-Lonergan E., Dekkers J.C.M., Gabler N.K., Lonergan S.M. (2013). Divergent genetic selection for residual feed intake impacts mitochondria reactive oxygen species production in pigs1. J. Anim. Sci..

[40] Banerjee P., Carmelo V.A.O., Kadarmideen H.N. (2020). Genome-Wide Epistatic Interaction Networks Affecting Feed Efficiency in Duroc and Landrace Pigs. Front. Genet..

[41] Higgins M.G., Fitzsimons C., McClure M.C., McKenna C., Conroy S., Kenny D.A., McGee M., Waters S.M., Morris D.W. (2018). GWAS and eQTL analysis identifies a SNP associated with both residual feed intake and GFRA2 expression in beef cattle. Sci. Rep..

[42] Bijvelds M.J.C., Tresadern G., Hellemans A., Smans K., Nieuwenhuijze N.D.A., Meijsen K.F., Bongartz J.-P., Ver Donck L., de Jonge H.R., Schuurkes J.A.J. (2018). Selective inhibition of intestinal guanosine 3′,5′-cyclic monophosphate signaling by small-molecule protein kinase inhibitors. J. Biol. Chem..

[43] Valente T.S., Baldi F., Sant’Anna A.C., Albuquerque L.G., Paranhos da Costa M.J.R. (2016). Genome-Wide Association Study between Single Nucleotide Polymorphisms and Flight Speed in Nellore Cattle. PLoS ONE.

[44] Valentino M.A., Lin J.E., Snook A.E., Li P., Kim G.W., Marszalowicz G., Magee M.S., Hyslop T., Schulz S., Waldman S.A. (2011). A uroguanylin-GUCY2C endocrine axis regulates feeding in mice. J. Clin. Investig..

[45] Rauw W.M., Portolés O., Corella D., Soler J., Reixach J., Tibau J., Prat J.M., Diaz I., Gómez-Raya L. (2007). Behaviour influences cholesterol plasma levels in a pig model. Animal.

[46] Zhang L., Rajbhandari P., Priest C., Sandhu J., Wu X., Temel R., Castrillo A., de Aguiar Vallim T.Q., Sallam T., Tontonoz P. (2017). Inhibition of cholesterol biosynthesis through RNF145-dependent ubiquitination of SCAP. Elife.

[47] Holeček M. (2001). Effect of starvation on branched-chain α-keto acid dehydrogenase activity in rat heart and skeletal muscle. Physiol. Res..

[48] Duan Y., Duan Y., Li F., Li Y., Guo Q., Ji Y., Tan B., Li T., Yin Y. (2016). Effects of supplementation with branched-chain amino acids to low-protein diets on expression of genes related to lipid metabolism in skeletal muscle of growing pigs. Amino Acids.

[49] Mukiibi R., Vinsky M., Keogh K., Fitzsimmons C., Stothard P., Waters S.M., Li C. (2019). Liver transcriptome profiling of beef steers with divergent growth rate, feed intake, or metabolic body weight phenotypes1. J. Anim. Sci..

[50] Sung Y.J., Pérusse L., Sarzynski M.A., Fornage M., Sidney S., Sternfeld B., Rice T., Terry J.G., Jacobs D.R., Katzmarzyk P. (2016). Genome-wide association studies suggest sex-specific loci associated with abdominal and visceral fat. Int. J. Obes..

[51] Valadkhan S., Gunawardane L.S. (2013). Role of small nuclear RNAs in eukaryotic gene expression. Essays Biochem..

[52] Sun J.S., Manley J.L. (1995). A novel U2-U6 snRNA structure is necessary for mammalian mRNA splicing. Genes Dev..

[53] Ravi S., Schilder R.J., Kimball S.R. (2015). Role of Precursor mRNA Splicing in Nutrient-Induced Alterations in Gene Expression and Metabolism. J. Nutr..

[54] Mansouri E., Khorsandi L., Moaiedi M.Z. (2015). Grape seed proanthocyanidin extract improved some of biochemical parameters and antioxidant disturbances of red blood cells in diabetic rats. Iran. J. Pharm. Res..

[55] Russell J.R., Sexten W.J., Kerley M.S., Hansen S.L. (2016). Relationship between antioxidant capacity, oxidative stress, and feed efficiency in beef steers. J. Anim. Sci..

[56] Anders S., Huber W. (2010). Differential expression analysis for sequence count data. Genome Biol..

[57] Ritchie M.E., Phipson B., Wu D., Hu Y., Law C.W., Shi W., Smyth G.K. (2015). limma powers differential expression analyses for RNA-sequencing and microarray studies. Nucleic Acids Res..

[58] Chong J., Soufan O., Li C., Caraus I., Li S., Bourque G., Wishart D.S., Xia J. (2018). MetaboAnalyst 4.0: Towards more transparent and integrative metabolomics analysis. Nucleic Acids Res..

[59] Mostafavi S., Ray D., Warde-Farley D., Grouios C., Morris Q. (2008). GeneMANIA: a real-time multiple association network integration algorithm for predicting gene function. Genome Biol..

[60] Eden E., Navon R., Steinfeld I., Lipson D., Yakhini Z. (2009). GOrilla: a tool for discovery and visualization of enriched GO terms in ranked gene lists. BMC Bioinform..

[61] Bindea G., Mlecnik B., Hackl H., Charoentong P., Tosolini M., Kirilovsky A., Fridman W.H., Pagès F., Trajanoski Z., Galon J. (2009). ClueGO: A Cytoscape plug-in to decipher functionally grouped gene ontology and pathway annotation networks. Bioinformatics.

