# Supplementary material for: Integrative Analysis of Metabolomic and Transcriptomic Profiles Uncovers Biological Pathways of Feed Efficiency in Pigs
Source: Metabolites. 2020 Jul 6;10(7):275. doi: 10.3390/metabo10070275 (PMC7408121; doi:10.3390/metabo10070275)
Supplement: Supplementary file 1 [file metabolites-10-00275-s001.zip › Figure S1.docx]

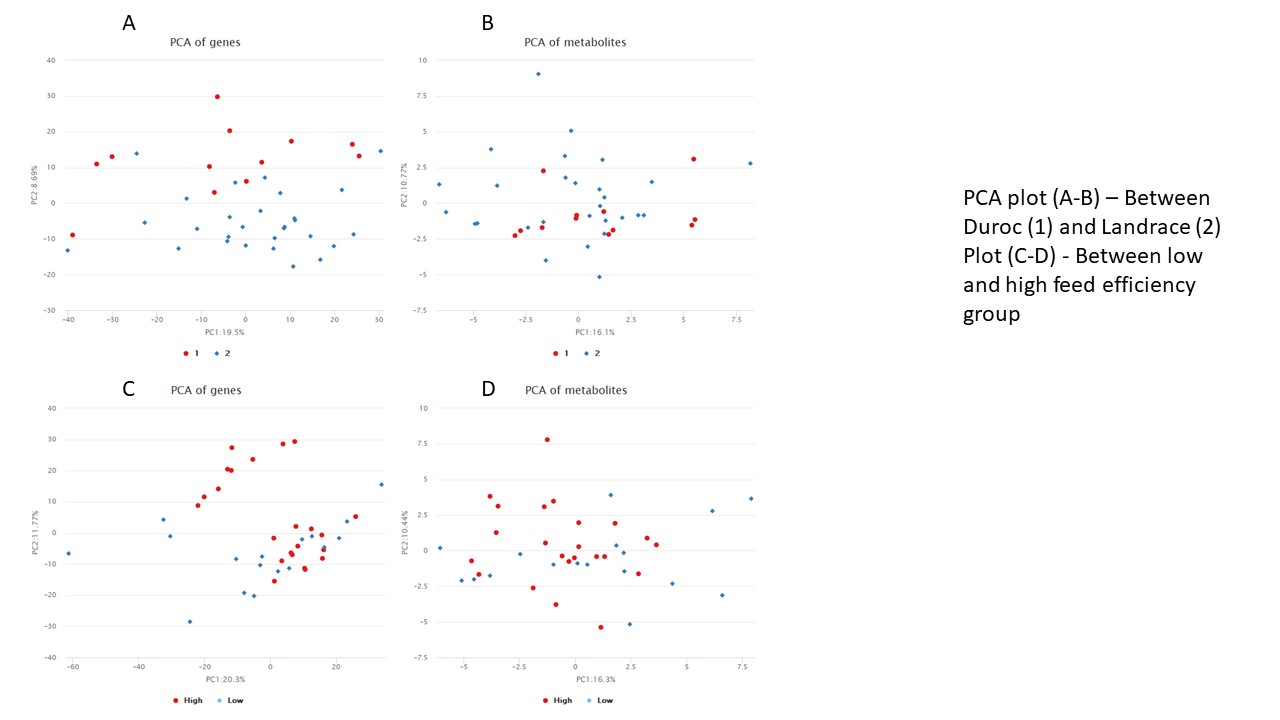


**Figure S1**. The principal component analysis of metabolites and genes. A and B: PCA plot of genes and metabolites, respectively, in (1) Duroc and (2) Landrace; C and D: PCA plot of genes and metabolites, respectively, in high and low feed efficient groups.
